# What does a professionalism assessment scale measure?

**DOI:** 10.5116/ijme.5539.e386

**Published:** 2015-05-04

**Authors:** Pieter Barnhoorn

**Affiliations:** Department of Public Health and Primary Care, Leiden University Medical Center, The Netherlands

Klemenc-Ketis and Vrecko described the development and validation of a professionalism assessment scale for medical students in a recent issue of this journal.^1^ In the introduction to this article they state that 'professionalism can be defined as a collection of attitudes, values, behaviours and relationships that act as the foundation of the health professional's contract with society'. Subsequently, the authors describe how they have developed a professionalism assessment scale for medical students. They used input from teachers as well as from students, because they argue that it is important to incorporate students' opinions about professionalism into research in this field.

The engagement of students in the study of professionalism is of great importance. The experts on professionalism do not have a monopoly on wisdom. However, there is someone who knows best, what is, meant by professionalism, namely, the patient. Professionalism can be defined as 'placing the best interests of patients at the centre of everything you do'.^2^ The patient is the sole reason for the existence of us doctors! In addition to incorporating students' opinions about professionalism it is necessary to incorporate patients' opinions on assessment scales in further research.

Furthermore, it is important to ask ourselves what we are measuring when we claim to measure professionalism. The items of the professionalism assessment scale measure beliefs about professionalism. The question is whether beliefs about professionalism are synonymous with professionalism. Beliefs drive behaviour. However, the actual demonstrated behaviour not only depends on beliefs but on other factors, such as the environment and on students' competencies. A helpful model, which can be used to differentiate between these levels, may be the onion model described by Korthagen.^3^ This model consists, of the following layers, from the outside to the inside: environment, behaviour, competencies, beliefs, identity and, at the centre mission. It can serve as a framework for reflection and development.^3^ The levels in this model can be seen from different perspectives. From each perspective, there will be a differet answer to the question of the essential qualities of a professional doctor, while it is also possible to employ various perspectives parallel to one another. Many a medical student values, for instance the empathy items, described by Klemenc-Ketis and Vrecko,^1^ but does that imply that they can behave empathetically in a stressful environment?

Incorporating patients opinions and differentiation between levels of professionalism might take this valuable research on professionalism assessment scale measure to a higher level.

## Conflict of Interest

The author declares that there are no conflicts of interest.
